# A Novel Pathogenic *BRCA1* Splicing Variant Produces Partial Intron Retention in the Mature Messenger RNA

**DOI:** 10.3390/ijms17122145

**Published:** 2016-12-21

**Authors:** Maria Valeria Esposito, Marcella Nunziato, Flavio Starnone, Antonella Telese, Alessandra Calabrese, Giuseppe D’Aiuto, Pietro Pucci, Massimiliano D’Aiuto, Francisco Baralle, Valeria D’Argenio, Francesco Salvatore

**Affiliations:** 1 CEINGE-Biotecnologie Avanzate, via Gaetano Salvatore 486, 80145 Naples, Italy; espositomari@ceinge.unina.it (M.V.E.); nunziato@ceinge.unina.it (M.N.); starnone@ceinge.unina.it (F.S.); telesea@ceinge.unina.it (A.T.); alessandra.underforty@gmail.com (A.C.); pucci@unina.it (P.P.); 2 Department of Movement Sciences and Wellness (DiSMEB), University of Naples Parthenope, via Medina 40, 80133 Naples, Italy; 3 Department of Senology, Istituto Nazionale Tumori–IRCCS Fondazione Pascale, via Mariano Semmola, 52, 80131 Naples, Italy; giuseppe@daiuto.org (G.D.); massimiliano.daiuto@gmail.com (M.D.); 4 International Centre for Genetic Engineering and Biotechnology, Science Park, Padriciano 99, 34149 Trieste, Italy; baralle@icgeb.org; 5 Department of Molecular Medicine and Medical Biotechnologies, University of Naples Federico II, via Sergio Pansini 5, 80131 Naples, Italy; 6 IRCCS-Fondazione SDN, via Emanuele Gianturco 113, 80143 Naples, Italy

**Keywords:** breast cancer, *BRCA1*, next generation sequencing, splicing mutation, partial intron retention

## Abstract

About 10% of all breast cancers arise from hereditary mutations that increase the risk of breast and ovarian cancers; and about 25% of these are associated with the *BRCA1* or *BRCA2* genes. The identification of *BRCA1*/*BRCA2* mutations can enable physicians to better tailor the clinical management of patients; and to initiate preventive measures in healthy carriers. The pathophysiological significance of newly identified variants poses challenges for genetic counseling. We characterized a new *BRCA1* variant discovered in a breast cancer patient during *BRCA1*/*2* screening by next-generation sequencing. Bioinformatic predictions; indicating that the variant is probably pathogenetic; were verified using retro-transcription of the patient’s RNA followed by PCR amplifications performed on the resulting cDNA. The variant causes the loss of a canonic donor splice site at position +2 in *BRCA1* intron 21; and consequently the partial retention of 156 bp of intron 21 in the patient’s transcript; which demonstrates that this novel *BRCA1* mutation plays a pathogenetic role in breast cancer. These findings enabled us to initiate appropriate counseling and to tailor the clinical management of this family. Lastly; these data reinforce the importance of studying the effects of sequence variants at the RNA level to verify their potential role in disease onset.

## 1. Introduction

Hereditary breast and ovarian cancers (HBOCs) account for about 10% of all breast cancers [1,2]. *BRCA1* and *BRCA2* germline mutations are the most frequent HBOC signatures, and they increase the lifetime risk of developing breast and ovarian cancers by as much as 80% [3,4]. About 1790 *BRCA1* and 2000 *BRCA2* variants are annotated in the Breast Cancer Information Core database (BIC, http://research.nhgri.nih.gov/bic/) and this number is expected to increase due to the diffusion of next-generation sequencing (NGS)-based screening of the *BRCA* genes. However, the clinical relevance of a large fraction of these variants remains unknown, and most *BRCA1* and *BRCA2* variants fall within the category of “unclassified variants” (UVs) [2,5,6].

It is important to understand the significance of *BRCA1* and *BRCA2* variants to ensure appropriate genetic counseling and clinical management of HBOC patients and their relatives [7]. Both functional assays and bioinformatics predictions have been used to evaluate the role of unclassified and novel *BRCA1* and *BRCA2* variants [8,9,10,11,12,13]. At the molecular level, a fraction of these variants occur within intronic regions; hence, it is conceivable that they affect splicing reactions and induce gene inactivation [14]. In the case of bioinformatics tools, a number of studies reported that bioinformatics predictions give reliable results only if the tested variants: (i) affect exon-intron junctions; (ii) generate new splice sites; or (iii) induce cryptic sites [11,12,13]. Specific in vitro functional assays, essentially based on hybrid minigenes, are effective in the analysis of splicing variants, especially when other blood samples cannot be obtained directly from the patients carrying the variants to be tested [9]. When sufficient blood samples can be obtained, analysis of the patient’s RNA is the most effective way to assess the pathogenicity of a specific variant that potentially induces splicing alterations [9].

Here, we report the characterization of a novel *BRCA1* splicing variant, identified by NGS, and demonstrate that it induces aberrant splicing, thus suggesting it plays a pathogenic role in increasing HBOC risk.

## 2. Results

**Figure 1:**
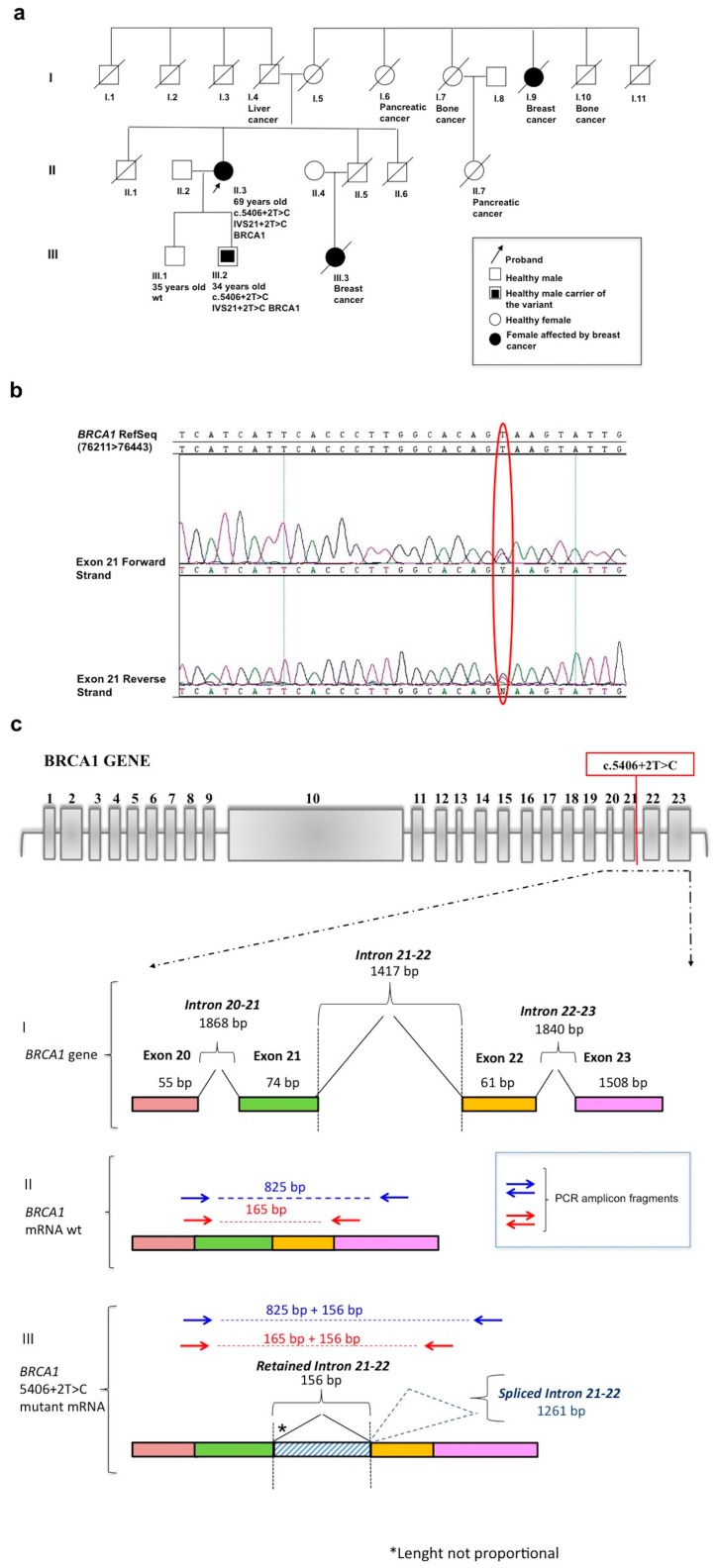
(**a**) The pedigree of the patient’s family, and the type of cancer in her deceased relatives; (**b**) The patient’s electropherogram. Exon 21 of the *BRCA1* gene (after next-generation sequencing) was analyzed by standard Sanger sequencing, which confirmed the presence of the c.5406+2T>C splice variant (encircled in red); (**c**) Schematic view of the effects of the variant. The schematic representation of the *BRCA1* gene exon-intron structure and the variant localization is reported in the top panel. Below is the scheme of the exon-intron sequence spanning from exon 20 to 23. The colored bars represent exons 20–23 of the *BRCA1* gene, at both the genomic (I) and cDNA level (II and III). The mutated cDNA carrying the retained 156 bp of intron 21 is shown (III). Arrows indicate the PCR amplicon fragments derived from the custom-designed primer pairs (see Materials and Methods).

The *BRCA1* variant c.5406+2T>C described herein was found in a 69-year-old woman, investigated for *BRCA* mutational status because she was affected by breast cancer (breast cancer onset age: 60 years), and because other tumors, including breast cancer, were present in her family, which suggested the presence of an HBOC syndrome (Figure 1a). NGS-based molecular testing of the patient did not reveal any known pathogenic mutation in either *BRCA1* or *BRCA2*. However, the patient carried a hitherto unknown intronic *BRCA1* variant, namely c.5406+2T>C (IVS21) (Figure 1b). This variant is not recorded in the BIC (http://research.nhgri.nih.gov/bic/), Ensemble (http://www.ensembl.org) or Human Genome Mutation (HGM, www.hgmd.cf.ac.uk) databases. Moreover, it was not identified in a large population-based study of 500 unrelated breast cancer patients, its allele frequency being 0.1%.

Unfortunately, we were not able to verify the segregation of the variant with the phenotype within the patient’s family because her parents and all affected relatives died before molecular testing. We were able to test the patient’s two sons (Figure 1a, III.1 and III.2). They are, respectively, 34 and 35 years old and are both healthy; the younger son carries the c.5406+2T>C mutation and has been enrolled in a surveillance program for cancer prevention.

To determine whether the *BRCA1* c.5406+2T>C variant is pathogenetic, we performed bioinformatic predictions using specific tools (see Materials and Methods). This in silico analysis suggested impairment of splicing reactions that probably induces the retention of *BRCA1* intron 21 (Figure 1c). In particular, the Human Splicing Finder results demonstrate the breakage of the wild type (wt) donor site and the possible use of a cryptic splice site. Moreover, the variant seems to generate many different potential exonic splicing enhancers from position 70 to 75, and the potential loss of one of these at position +2 on cDNA could create other sites. All these molecular events probably generate an alternative splicing process (see Supplementary Materials File S1).

We analyzed the patient’s RNA to verify the pathogenicity of this splicing variant as suggested by the bioinformatics predictions. Her cDNA was amplified using two primer pairs designed to test, at a molecular level, the *BRCA1* intron 21 retention, as described under Methods (Table S1). Two RNA samples obtained from women screened for *BRCA1*/*2* and carrying no mutations served as controls. DNA and RNA samples were also obtained from six healthy controls and were analyzed with the patient’s sample. All healthy controls were screened for the c.5406+2T>C variant, and were found to be non-carriers.

**Figure 2:**
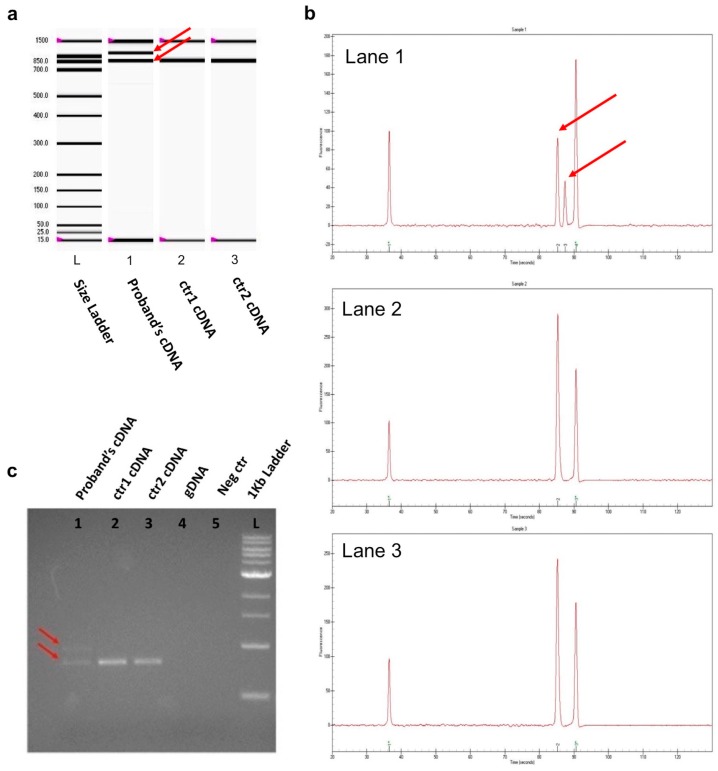

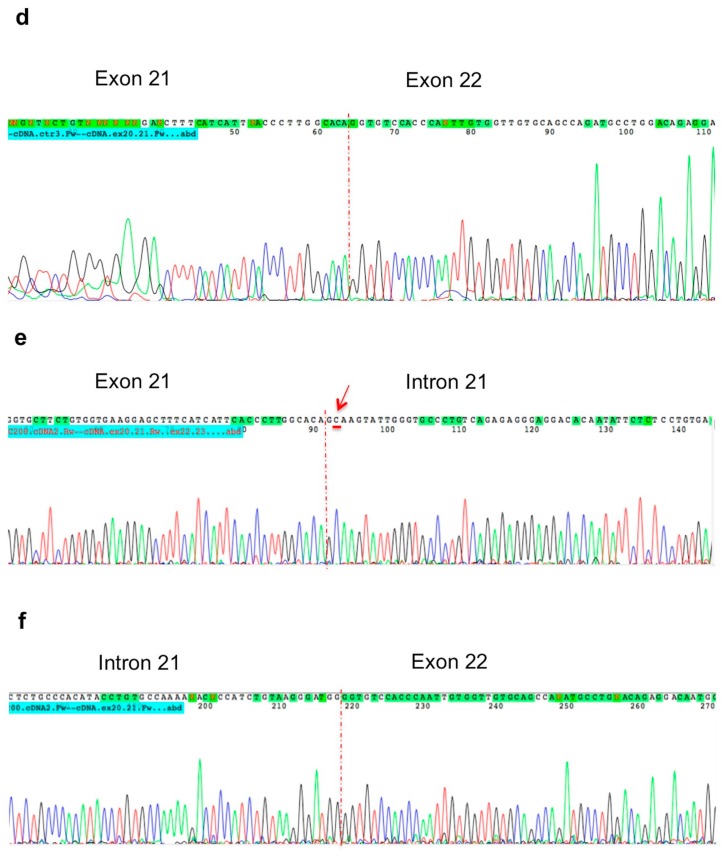
(**a**) DNA Chip 1K assay of the 825 bp amplification of the cDNAs of the patient and of the two women without *BRCA1* and *BRCA2* mutations (primers a and b). Lane 1 was loaded with the mutated cDNA that gave two amplification products: the 825 bp band represents the wt *BRCA1* allele, while the intronic retention of mutated cDNA generated the other band, which was 156 bp longer than wt. Lanes 2 and 3 show the *BRCA1*-amplified cDNA of the two control subjects (cDNA ctr1 and cDNA ctr2); (**b**) Peak view of the fluorescence results; (**c**) PCR amplifications to verify the splicing variant visualized on agarose gel at 1.2%. Lanes 1–5 show the results of the amplification on cDNA templates. Lane 1 shows the two bands derived from amplification of the mutated cDNA of the patient (825 and 981 bp); lanes 2 and 3 show the amplifications performed on the cDNA of the two women without *BRCA1* and *BRCA2* mutations; lane 4 was loaded with gDNA which shows that no products were generated consistent with the PCR reaction negative control (neg ctr) loaded in lane 5; lane L was loaded with 7 µL of size marker 1 kb; the red arrows show the *wt* and the mutated alleles, containing the intron retention; (**d**) Sanger sequencing of a healthy control cDNA showing exons 21 and 22 of the *BRCA1* gene; (**e**) Sequence of the cDNA of the patient bearing the c.5406+2T>C splice variant (red arrow) showing the junctions between exon 21 and intron 21; and (**f**) between intron 21 and exon 22, hence the retention of 156 bp of intron 21.

A primer pair was designed on the cDNA sequence to anneal on the 20/21 exon junction (forward primer) and on exon 23 (reverse primer). The expected amplification product size was 825 bp (Table S1a,b), as predicted by Primer Blast. All cDNA samples obtained from the patient and controls showed an amplification product of 825 bp, which confirmed they were successfully retro-transcribed. The mutated cDNA produced an additional amplicon of 981 bp (Figure 2a–c). To avoid genomic DNA contamination, a second amplification was carried out using a primer pair that covered a 165 bp region on the *BRCA1* cDNA, annealing on the 20/21 and 22/23 exon junctions (Table S1c,d). In this case, an additional band 156 bp longer than 165 bp, corresponding to the mutated allele, appeared in the DNA of the proband and her son, both of whom are mutation carriers (data not shown). All six healthy controls showed only one band of 165 bp, corresponding to the expected product amplification of the wt alleles.

To verify these findings, we analyzed all PCR products by standard Sanger sequencing (Figure 2d–f. The healthy control cDNAs contained exon 21 followed by exon 22 of the *BRCA1* gene (Figure 2d). We used Sanger sequencing to search for the intronic 156 bp: the junction sequences between exon 21 and the 156 bp of the retained intron 21 of the *BRCA1* gene (Figure 2e), as well as the intron sequence joined to exon 22 (Figure 2f) in the patient and in her son. The resulting sequences of the wt and mutated cDNA are also reported in the FASTA format (Figure S1).

## 3. Discussion

An understanding of the significance of *BRCA1* and *BRCA2* variants enables appropriate genetic counseling and clinical management of HBOC patients and their relatives [7]. A major limitation of *BRCA1* and *BRCA2* genetic testing, especially after the diffusion of NGS-based screening, is the number of variants of unknown significance that pose clinical challenges. Mutations in the splicing site junction can lead to altered splicing and thus adversely affect the translated protein [15]. In this context, RNA analyses, preferably conducted on the patient’s blood samples, should be used to verify the pathogenicity of specific sequence variations.

Here, we report the functional evaluation of a novel *BRCA1* splicing variant, namely c.5406+2T>C (IVS21+2T>C). The in silico analysis predicted that this variant exerts a pathogenic effect. Our data obtained on the patient’s RNA showed that the tested variant affects the splicing reactions and results in the retention of 156 bp of intron 21.

In this context, Steffensen et al. reported a *BRCA1* c.5406+1G>A variant that also causes a splicing anomaly [16]. This variant, which is located just one nucleotide before the variant that we describe herein, causes exon 22 skipping consequent to the loss of the wt donor site in position +1, whereas variant c.5406+2T>C, in addition to the loss of the canonic donor splice site, creates a new splice site that results in the retention of 156 bp of intron 21. This is in line with the previous finding that the GC dinucleotide is an efficient 5′ donor site and that spliceosomal recognition may facilitate the insertion of the cryptic exon and not the skipping [17]. In addition, the alternative 5′ splice site in our mutated allele scores as a rather good site because it is predicted to be 0.83 by the splice spite predictor tool [18]. However, the configuration of the GTAAGGGATGG/GTAAGGATT sequence shows that the U1 binding site of the alternative 5′ splice site may be a binding site for hnRNPh/F and would hamper the recognition of the splice site, thereby accounting for the low level of mutant mRNA found in the cells [19]. Furthermore, it is widely recognized that splicing alterations are a common consequence of *BRCA1* variants [8,9,10,11,12,16].

The retention of 156 bp of intron 21 enables the maintenance of the reading frame and the translation of a putative BRCA1 protein 55 amino acids longer than the wt protein. The mutation in the BRCT (BRCA1 C Terminus) domain of the BRCA1 protein could impair the folding of and/or binding to proteins such as P53, BRIP1, BACH1, Abraxas and CtIP, which are necessary for the activation and the proper functioning of the DNA double-strand break repair pathway which governs the maintenance of genome integrity (Figure S2) [20]. In addition, the lower level of mRNA derived from the mutant allele could, in any case, result in lower levels of active protein, and the protein with the additional 55 amino acids may be degraded by the cell’s surveillance systems. Indeed, the mRNA carrying fragments of intron can be degraded in the nucleus and/or poorly exported to the cytoplasm [21]. Thus, evaluation of the effects of c.5406+2T>C on the BRCA1 protein could shed light on these aspects. However, given the low level of mRNA and the translated protein, the resolution of the Western blot does not easily reveal the mutated protein. To overcome this limitation, we are evaluating whether mass spectrometry may be a more sensitive method with which to distinguish cleaved BRCA1 mutated peptides from cleaved wild-type peptides.

Another limitation of this study is the inability to verify the segregation of the variant within the family, since the patient’s ancestors and the many affected relatives are deceased. In addition, the only other carrier of the variant, being male and still young, is uninformative in terms of pathogenicity. The male risk of *BRCA* mutations is lower than the female risk and late penetration cannot be excluded [22]. Moreover, we routinely carry out NGS-based molecular screening of the *BRCA1/2* genes and, among the more than 1300 alleles analyzed to date, we have not identified the *BRCA1* c.5406+2T>C (IVS21+2T>C) in any other patients.

Intron retention, which is a molecular phenomenon described in many diseases, is a consequence of single nucleotide variations at crucial splice control points such as donor or acceptor sites [21]. The aberrant splicing deriving from these gene variants can result in partial or full retention of specific introns, thereby leading to inactivation of tumor suppressor genes and thus to tumorigenesis. Indeed, this process is commonly observed in various neoplasias, including breast cancer [23].

Here, we describe a novel *BRCA1* splicing variant that could increase the risk of HBOC. Besides identifying the cancer-predisposing mutation in our HBOC family and defining its functional effect, we also identified an at-risk healthy member of this family, who can therefore start an ad hoc prevention program, bearing in mind that a male mutation carrier is also at risk of developing breast cancer. Although further experiments are needed to clarify the molecular effects exerted by the c.5406+2T>C splice variant on the BRCA1 protein, the results reported herein could explain the pathogenicity of this novel mutation, particularly in view of the various cases of cancer in the analyzed family. These data reinforce the importance of studying the effects of sequence variants at the RNA level to verify their potential role in disease onset.

## 4. Materials and Methods

### 4.1. Patients 

Patients were enrolled among women attending the Breast Unit of the “Istituto Nazionale dei Tumori—Fondazione G. Pascale” of Naples, Italy. Patients’ sons were available for evaluation. All patients gave their written informed consent to the study prior to blood sampling.

### 4.2. DNA Extraction and Molecular Screening of the BRCA Genes

Genomic DNA was obtained from peripheral blood samples using the Nucleon BACC3 Genomic DNA Extraction Kit (GE Healthcare, Little Chalfont, UK), according to the manufacturer’s instructions. *BRCA1*/*2* molecular analysis was carried out using a NGS-based method as previously reported [24]. Briefly, amplicon DNA libraries were prepared for each sample to analyze all the *BRCA1*/*2* coding exons and their flanking regions, according to manufacturer’s instructions (Multiplicom, Niel, Belgium). Sequencing reactions were carried out on the MiSeq instrument (250 × 2 PE, Illumina, CA, USA). The SeqPilot software (version 3.5.2, JSI Medical Systems, Tullastr, Germany) was used to analyze the NGS sequence data. Pathogenic mutations were confirmed by Sanger sequencing.

The nomenclature of the DNA variant is based on the *BRCA1* cDNA sequence (Ensembl: ENST00000357654), according to the recommendations of the Human Genome Variation Society (HGVS, http://www.hgvs.org/).

### 4.3. Bioinformatic Analysis

Human Splicing Finder (http://www.umd.be/HSF/, Marseille, France) and NetGene2 (http://www.cbs.dtu.dk/services/NetGene2/, Lyngby, Denmark) softwares were used to in silico evaluate the possible effects of the identified variant on gene splicing. Splice site scores were evaluated with the ‘Splice Site Prediction by Neural Network Site’ software (http://www.fruitfly.org/seq_tools/splice.html, Berkeley, CA, USA).

### 4.4. RNA Extraction and RT-PCR Analysis

Total RNA was isolated using the TRIzol^®^ protocol (Life Technologies, Carlsbad, CA, USA) and retro-transcribed using the High Capacity cDNA Reverse Transcription Kit (Applied Biosystems, Foster City, CA, USA), according to the manufacturer’s instructions. The cDNAs were amplified using primer pairs specifically designed as follows: (i) a primer pair spanning *BRCA1* exons 21 and 22 to include the region containing the splicing variant; in particular, the forward primer was designed on the 20/21 exon junction and the reverse primer annealed on exon 23, giving an amplification of 825 bp; and (ii) a primer pair amplifying 165 bp, with the forward primer complementary to 20/21 exon junction and the reverse primer annealing to 22/23 exon junction of the *BRCA1* gene (Table S1). The specificity of both primer pairs was verified by using Primer Blast (www.ncbi.nlm.nih.gov/tools/primer-blast/). PCR reactions were carried out using the 5 PRIME MasterMix (Eppendorf, Hamburg, Germany), with the following conditions: 94 °C for 2 min, 94 °C for 40 s, 60 °C (for the first pair) and 62 °C (for the second pair) for 40 s and 65 °C for 40 s for 40 cycles, 65 °C for 10 min. Agarose gels, the DNA Chip 1 K (BioRad, Hercules, CA, USA) and the High Sensitivity D1000 ScreenTape (Agilent Technologies, Santa Clara, CA, USA) were used to verify the amplification products. Next, Sanger sequencing was performed with an ABI 3130 capillary sequencer (Applied Biosystems Inc., Foster City, CA, USA). Sequences analysis was carried out with the SeqMan (DNASTAR, Inc., Madison, WI, USA) and CodonCode Aligner V3.5.7 (CodonCode Co., Centerville, MA, USA) tools.

## 5. Conclusions

Correct interpretation of the DNA sequence variants identified during NGS molecular testing is critical for correct diagnosis, risk stratification of patients and/or clinical decision-making [25]. The case described herein highlights the benefit of using NGS for *BRCA* gene testing. Using this technology, we were able to detect a previously unreported variant, to verify its potential pathogenetic effect, and to better define the HBOC risk in the patient and her family. The integration of NGS for high-throughput DNA scanning with other molecular and cellular techniques for the assessment of the significance of variants will improve the sensitivity of molecular diagnostics as a whole, thereby leading to even more personalized medicine.

## Supplementary Materials

Supplementary materials can be found at www.mdpi.com/1422-0067/17/12/2145/s1.

## Abbreviations

HBOCs	Hereditary breast and ovarian cancers
BIC	Breast Cancer Information Core database
UV	Unclassified Variants
NGS	Next Generation Sequencing
