# Supplementary material for: A Novel Pathogenic BRCA1 Splicing Variant Produces Partial Intron Retention in the Mature Messenger RNA
Source: Int J Mol Sci. 2016 Dec 21;17(12):2145. doi: 10.3390/ijms17122145 (PMC5187945; doi:10.3390/ijms17122145)
Supplement: Supplementary file 1 [file ijms-17-02145-s001.zip › File S1.pdf]

**Supplementary Table 1:** Human Splicing Finder Output. The tables show the results obtained with Human Splicing Finder software performed on the DNA sequence bearing the c.5406+2T>C splice variant.

BRCA1 c.5406+2T>C

**Sequences**

**Reference sequence**

| | |
|---|---|
| BRCA1 Gene > ENST00000357654 Transcript > Exon number: 21 (74 bp) + 100 intronic nucleotides at exon ends | |
| 1 | aggtcttgct ataagccttc atccggagag tgtagggtag agggcctggg ttaagtatgc agattactgc agtgatttta catctaaatg tccattttag |
| 101 | ATCAACTGGA ATGGATGGTA CAGCTGTGTG GTGCTTCTGT GGTGAAGGAG CTTTCATCAT TCACCCTTGG CACAgtaagt attgggtgcc ctgtcagaga |
| 201 | gggaggacac aatattctct cctgtgagca agactggcac ctgtcagtcc ctatggatgc ccctactgta gcct |

Total sequence length: 274 nucleotides

## Mutant sequence

| | |
|---|---|
| 1 | aggtcttgct ataagccttc atccggagag tgtagggtag agggcctggg ttaagtatgc agattactgc agtgatttta catctaaatg tccattttag |
| 101 | ATCAACTGGA ATGGATGGTA CAGCTGTGTG GTGCTTCTGT GGTGAAGGAG CTTTCATCAT TCACCCTTGG CACAgcaagt attgggtgcc ctgtcagaga |
| 201 | gggaggacac aatattctct cctgtgagca agactggcac ctgtcagtcc ctatggatgc ccctactgta gcct |

Total sequence length: 274 nucleotides

The underlined sequences are analyzed by HSF.

### Interpreted Data

This table shows only relevant results related to the mutation position and context.

| Predicted signal | Prediction algorithm | cDNA Position | Interpretation |
|---|---|---|---|
| Broken WT Donor Site | 1 - HSF Matrices<br>2 - MaxEnt | 12GGCACAgtaagtattgggtgc cctgtcaga70727476788082 | Alteration of the WT donor site,<br>most probably affecting splicing. |

### Raw Data Tables

In the tables below, positions in sequence for the 5' intron are labeled as negative and as positive for the 3' intron.
Variations in the tables below are noted in colored boxes, according to the following scale:

| Site broken | 0% - 25% variation | 26% - 50% variation | 51% - 75% variation | 76% - 100% variation | New site< |
|---|---|---|---|---|---|

[Potential Splice Sites]
[Potential Branch Points]
[Enhancer motifs]
[Silencer motifs]
[Other splicing motifs]

## HSF Matrices

| Sequence Position | cDNA Position | Splice site type | Motif | New splice site | Wild Type | Mutant | If cryptic site use, exon length variation | Variation (%) |
|---|---|---|---|---|---|---|---|---|
| 164 | 64 | Acceptor | CCCTTGGCACAgta | cccttggcacagCA | 82.96 | 83.1 | NA | +0.17 |
| 168 | 68 | Acceptor | TGGCACAgtaagta | tggcacagcaagTA | 67.31 | 67.97 | NA | +0.98 |
| 172 | 72 | Donor | ACAgtaagt | ACAgcaagt | 83.72 | 56.88 | 83 | WT site broken -32.06 |
| 176 | 2 | Donor | taagtattg | CAAgtattg | 64.39 | 66.37 | NA | New site +3.08 |

## MaxEnt

Threshold values:

5' Motif: 3
3' Motif: 3

| Sequence Position | cDNA Position | 5' Motif | | | | | 3' Motif | | | | |
|---|---|---|---|---|---|---|---|---|---|---|---|
| | | Ref Motif | Ref Score | Mut Motif | Mut Score | Variation (%) | Ref Motif | Ref Score | Mut Motif | Mut Score | Variation (%) |
| 156 | 56 | | | | | | ATCATTCACCCTTGGCACAg taa | 2.97 | atcattcacccttggcacagCAA | 3.56 | +219.87 |
| 172 | 72 | ACAgtaagt | 9.49 | ACAgcaagt | 1.74 | -81.66 | | | | | |

## Branch Points calculation is performed using a new algorithm.

No difference between mutant and reference sequence was found with this matrice.

## ESE Finder matrices for SRp40, SC35, SF2/ASF and SRp55 proteins

Threshold values:

SF2/ASF: 72.98
SF2/ASF (IgM-BRCA1): 70.51
SRp40: 78.08
SC35: 75.05

SRp55: 73.86

Variation expresses the difference between reference and mutant values. Wild Type value is taken as reference.

| Sequence Position | cDNA Position | Linked SR protein | Reference Motif (value 0-100) | Linked SR protein | Mutant Motif (value 0-100) | Variation |
|---|---|---|---|---|---|---|
| 170 | 70 | | | SRp40 | GCACAgc (79.94) | New site |
| 171 | 71 | SF2/ASF (IgM-BRCA1) | CACAgta (84.23) | SRp55 | CACAgc (77.77) | -7.68 % |
| 171 | 71 | SF2/ASF (IgM-BRCA1) | CACAgta (84.23) | SF2/ASF (IgM-BRCA1) | CACAgca (83.46) | -0.91 % |
| 171 | 71 | SF2/ASF (IgM-BRCA1) | CACAgta (84.23) | SF2/ASF | CACAgca (81.48) | -3.27 % |
| 171 | 71 | SF2/ASF | CACAgta (84.10) | SRp55 | CACAgc (77.77) | -7.53 % |
| 171 | 71 | SF2/ASF | CACAgta (84.10) | SF2/ASF (IgM-BRCA1) | CACAgca (83.46) | -0.76 % |
| 171 | 71 | SF2/ASF | CACAgta (84.10) | SF2/ASF | CACAgca (81.48) | -3.12 % |
| 176 | 2 | SRp55 | taagta (76.10) | | | Site broken -100 |

## RESCUE ESE hexamers

| Sequence Position | cDNA Position | Enhancer motif reference sequence | Enhancer motif mutant sequence | Variation |
|---|---|---|---|---|
| 176 | 2 | | caagta | New Site |

## Predicted PESE Octamers from Zhang & Chasin

No difference between mutant and reference sequence was found with this matrice.

## EIEs from Zhang et al.

| Sequence Position | cDNA Position | Enhancer motif reference sequence | Enhancer motif mutant sequence | Variation |
|---|---|---|---|---|
| 173 | 73 | | CAgcaa | New Site |
| 174 | 74 | | Agcaag | New Site |
| 175 | 75 | | gcaagt | New Site |

## ESE motifs from HSF - Experimental

No difference between mutant and reference sequence was found with this matrice.

## Silencer motifs from Sironi et al.

No difference between mutant and reference sequence was found with this matrice.

## ESS decamers from Wang et al.

No Silencer motif found with this matrice

### Fas-ESS hexamers

| Sequence Position | cDNA Position | Reference sequence | Set | Mutant sequence | Set | Variation |
|---|---|---|---|---|---|---|
| 75 | 75 | gtaagt | 2, 3 | | | Site broken |

### PESS Octamers from Zhang & Chasin

No Silencer motif found with this matrice

### IIEs from Zhang et al.

No difference between mutant and reference sequence was found with this matrice.

### hnRNP motifs - Experimental

Threshold values:

hnRNP A1: 65.476

Variation expresses the difference between reference and mutant values. Wild Type value is taken as reference.

| Sequence Position | cDNA Position | Linked hnRNP protein | Reference Motif (value 0-100) | Linked hnRNP protein | Mutant Motif (value 0-100) | Variation |
|---|---|---|---|---|---|---|
| 176 | 2 | hnRNP A1 | taagta (71.19) | | | Site broken -100 |

### Exonic Splicing Regulatory Sequences from Goren et al.

| Sequence Position | cDNA Position | Reference motif | Mutant motif | Variation |
|---|---|---|---|---|
| 174 | 74 | | Agcaag | New Site |
| 176 | 2 | | caagta | New Site |

2013/2014 © Human Splicing Finder - Designed by Ghadi Rai - All rights reserved
Inserm UMR_S910 - Aix Marseille Université, 27 Boulevard Jean Moulin, 13385 Marseille Cedex 05
Tables interpretation guideline - HSFclose

## Types of matrices available in HSF

Sequences involved in splicing can be searched by either an exact motif approach or a degenerated consensus motif approach.

The exact motif approach implies that HSF searches for motifs present in its database. It finds them on the sequence or not.

The degenerated consensus motif approach is proceeded with Position Weight Matrices (PWM). PWM allow to give a specific weight to each position (nucleotide) of a sequence independantly from other nucleotides, allowing to detect consensus sequences (as far as main nucleotides are conserved). When the sum of all scores reaches or goes above a given threshold (fixed either by HSF or by user), HSF considers the analyzed sequence to be a potential motif for the aforementionned protein.

## Table fields

**Position**: Position on the analyzed sequence of the first nucleotide of the motif

**Linked protein**: If the motif is linked to a specific protein, it is noted here
**Motif**: The motif on the analyzed sequence, respecting exon and intron junctions
**Consensus value**: Please see below.

## Consensus value, thresholds and variations

**Consensus value**: This value is calculated by making the sum of all scores plus a constant.
**Threshold**: This value is a detection limit. Above or equal to it, a CV is considered to correspond to a motif. Under it, a CV correspond to nothing.
**Variation**: This value is different for "Analyze a sequence" or "Analyze mutation(s)" sections. In "Analyze a sequence" calculation, this value represents the strength of the motif (difference between the motif's CV and its tld). In "Analyze mutation(s)" calculation, this value represents the difference between the reference (WT) and mutant motif's strengths.
