# Supplementary material for: A Novel Pathogenic BRCA1 Splicing Variant Produces Partial Intron Retention in the Mature Messenger RNA
Source: Int J Mol Sci. 2016 Dec 21;17(12):2145. doi: 10.3390/ijms17122145 (PMC5187945; doi:10.3390/ijms17122145)
Supplement: Supplementary file 1 [file ijms-17-02145-s001.zip › ijms-161002-suppl.pdf]

# Supplementary Materials: A Novel Pathogenic *BRCA1* Splicing Variant Produces Partial Intron Retention in the Mature Messenger RNA

**Maria Valeria Esposito, Marcella Nunziato, Flavio Starnone, Antonella Telese, Alessandra Calabrese, Giuseppe D'Aiuto, Pietro Pucci, Massimiliano D'Aiuto, Francisco Baralle, Valeria D'Argenio and Francesco Salvatore**

**a**

```
ATCTTCAGGGGGCTAGAAATCTGTTGCTATGGGCCCTTCACCAACATGCCCACAGATCAA
CTGGAATGGATGGTACAGCTGTGTGGTGCTTCTGTGGTGAAGGAGCTTTCATCATTCACC
CTTGGCACAGGTGTCCACCCAATTGTGGTTGTGCAGCCAGATGCCTGGACAGAGGACAAT
GGCTTCCATGCAATTGGGCAGATGTGTGAGGCACCTGTGGTGACCCGAGAGTGGGTGTTG
GACAGTGTAGCACTCTACCAGTGCCAGGAGCTGGACACCTACCTGATACCCCAGATCCCC
CACAGCCACT
```

**b**

```
ATCTTCAGGGGGCTAGAAATCTGTTGCTATGGGCCCTTCACCAACATGCCCACAGATCAA
CTGGAATGGATGGTACAGCTGTGTGGTGCTTCTGTGGTGAAGGAGCTTTCATCATTCACC
CTTGGCACAgcaagtattgggtgccctgtcagagagggaggacacaatattctctcctgt
gagcaagactggcacctgtcagtccctatggatgcccctactgtagcctcagaagtcttc
tctgcccacatacctgtgccaaaagactccatctgtaagggatggGGTGTCCACCCAAT
TGTGGTTGTGCAGCCAGATGCCTGGACAGAGGACAATGGCTTCCATGCAATTGGGCAGAT
GTGTGAGGCACCTGTGGTGACCCGAGAGTGGGTGTTGGACAGTGTAGCACTCTACCAGTG
CCAGGAGCTGGACACCTACCTGATACCCCAGATCCCCCACAGCCACT
```

**Figure S1.** (**a**) FASTA sequence of a *wt* cDNA spanning from exon 20 to 23: exon 20 in yellow, 21 in green, 22 in violet and 23 in blue; (**b**) FASTA sequence of the cDNA of the patient bearing the c.5406+2T>C splice variant (in lower case and in red) showing the 156 retained bases of intron 21: exon 20 in yellow, 21 in green, 22 in violet and 23 in blue; the retained intron sequence is reported in lower case and white.

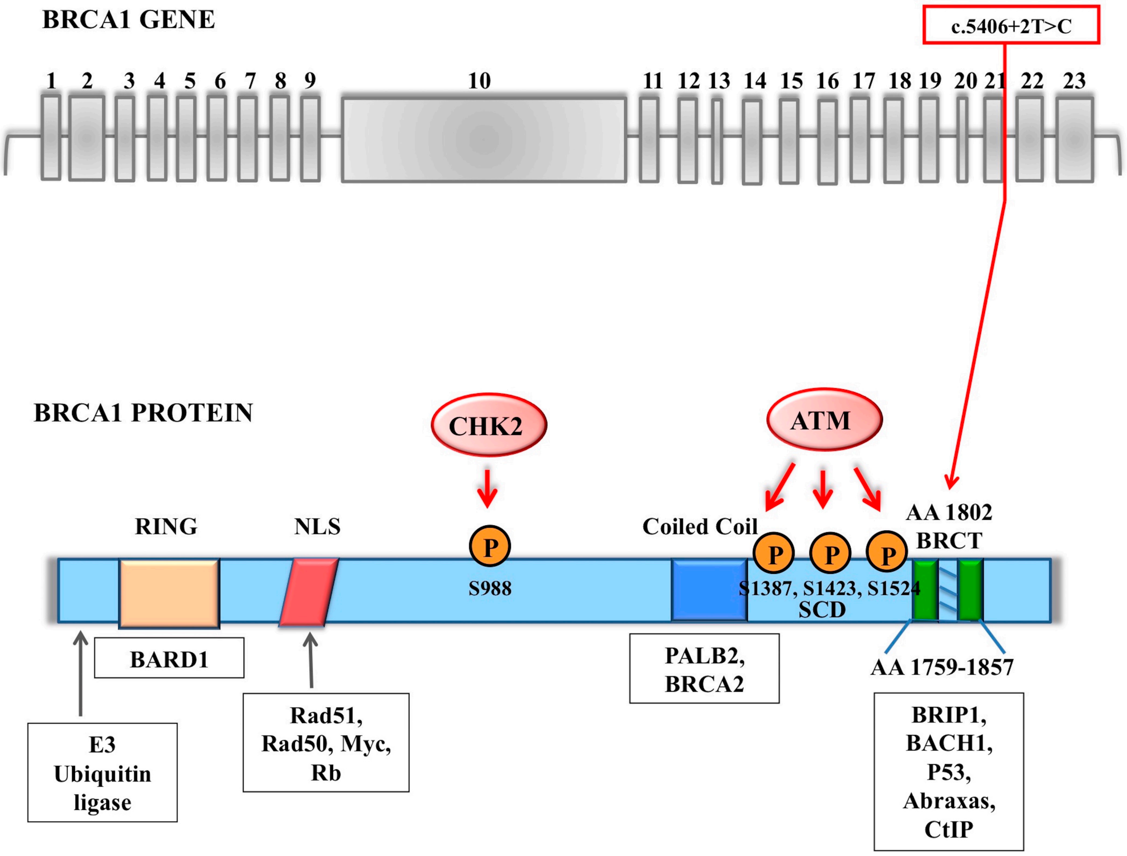


**Figure S2.** Schematic view of the BRCA1 protein and its interacting proteins. The function of the 3′-end proximity BRCT domain could be impaired by the c.5406+2T>C splice variant consequent to the altered repair of the double-strand break. The cross-talk with other proteins that interfere with the function of BRCA1 are indicated in boxes and the activating kinases are indicated by pink ovals and arrows.

**Table S1.** Primers used to assess the sequence regions of the novel *BRCA1* splicing variant (red and blue arrows correspond to those in Figure 1c).

| | Primer Sequences on BRCA1 cDNA | Product Length (bp) | |
|---|---|---|---|
| | | Wild Type | 5406+2T>C |
| **Blue arrows** | (a) BRCA1_cDNA_Fw: CAACATGCCCACAGATCAAC<br>(b) BRCA1_cDNA_Rw: AATTTCCTCCCCAATGTTCC | 825 | 981 |
| **Red arrows** | (c) cDNA_ex20/21_Fw: CAACATGCCCACAGATCAACTGGAATGG<br>(d) cDNA_ex22/23_Rw: CATCTGCCCAATTGCATGGAAGCCATTG | 165 | 321 |
